# Estimation of the Edge Crush Resistance of Corrugated Board Using Artificial Intelligence

**DOI:** 10.3390/ma16041631

**Published:** 2023-02-15

**Authors:** Tomasz Garbowski, Anna Knitter-Piątkowska, Jakub Krzysztof Grabski

**Affiliations:** 1 Department of Biosystems Engineering, Poznan University of Life Sciences, Wojska Polskiego 50, 60-627 Poznań, Poland; 2 Institute of Structural Analysis, Poznan University of Technology, Piotrowo 5, 60-965 Poznań, Poland; 3 Institute of Applied Mechanics, Poznan University of Technology, Jana Pawła II 24, 60-965 Poznań, Poland

**Keywords:** corrugated board, edge crush resistance, artificial intelligence, artificial neural network, deep learning, Gaussian processes

## Abstract

Recently, AI has been used in industry for very precise quality control of various products or in the automation of production processes through the use of trained artificial neural networks (ANNs) which allow us to completely replace a human in often tedious work or in hard-to-reach locations. Although the search for analytical formulas is often desirable and leads to accurate descriptions of various phenomena, when the problem is very complex or when it is impossible to obtain a complete set of data, methods based on artificial intelligence perfectly complement the engineering and scientific workshop. In this article, different AI algorithms were used to build a relationship between the mechanical parameters of papers used for the production of corrugated board, its geometry and the resistance of a cardboard sample to edge crushing. There are many analytical, empirical or advanced numerical models in the literature that are used to estimate the compression resistance of cardboard across the flute. The approach presented here is not only much less demanding in terms of implementation from other models, but is as accurate and precise. In addition, the methodology and example presented in this article show the great potential of using machine learning algorithms in such practical applications.

## 1. Introduction

The increasingly demanding packaging market forces manufacturers to provide solutions that ensure not only the ease of shaping the packaging, but also its sufficient strength and attractive appearance in the case of shelf-ready packaging (SRP) retail-ready packaging (RRP). Moreover, in the light of the growing global environmental awareness, there is an emphasis on materials that can be recycled, biodegraded and easily disposed of. Corrugated cardboard and packaging made from it fit perfectly into this trend and are widely utilized in many branches of industry, for instance, by cosmetics, food [1], transportation [2,3], agriculture [4] or furniture [5] companies.

Corrugated board is a type of sandwich structure with individual alternating layers consisting of flat and corrugated papers [6], the most often used cardboards ranging from two to seven layers. The profile of the corrugated layer, called fluting, is classified by the letters A (the tallest flute height), B, C, E, and F (the lowest flute height). The layered structure of the corrugated board results in two characteristic in-plane directions of orthotropy impacting on the mechanical strength of the cardboard, namely, the machine direction (MD) perpendicular to the main axis of the fluting and parallel to the paperboard fiber alignment, and cross direction (CD), which is parallel to the fluting.

When designing the corrugated cardboard packaging, it is crucial that they meet a certain load-bearing capacity, which is directly related to the mechanical properties of the paperboard, bearing in mind that the paper is an anisotropic material whose properties depend on the fibers alignment. The ease of shaping, which is considered a great advantage of the cardboard, may reduce the load capacity of the packaging, e.g., by introducing the ventilation holes, perforations and indentations [3,7,8,9,10] or creases [11,12]. Another factors that can diminish the strength of the cardboard are storage time, humidity and temperature [13,14] or stacking load [5,15,16]. The recycled or kraft papers, which can be applied in the corrugated cardboard lowering the cost, have the reduced mechanical parameters. Therefore, the optimal selection of the composition of the corrugated cardboard layers is of great importance for the load capacity of the packaging [17,18]. The strength of paper and corrugated board is affected also by geometrical and material imperfections [19,20].

In the light of the above, determining the material parameters and strength of the cardboard is a very demanding task, and the paper industry strives for effective, economical, straightforward solutions that, consequently, lead to the dynamic development of scientific research in this direction. Over the years, starting in the 1950s, the analytical formulas for typical box design have been developed [21,22,23], subsequently expanding into more complex approaches [7,19,24,25,26,27,28,29,30,31]. Hybrid [7,10,24,25,32,33] or purely numerical approaches [3,34,35] with the implementation of the finite element method (FEM) [2,3,36,37,38] are widely applicable as well. Considering the layered structure of the cardboard and the anisotropy of the paper, performing numerical analysis is a challenging task since the knowledge of material properties of each layer is a precondition. The procedure of reducing a multi-layered system to a single layered is called homogenization. This technique can be applied while using analytical [39,40] or numerical approach [8,34,41,42].

To unify the procedures of designating the mechanical properties of corrugated board standard tests have been developed. The most widespread are the box compression test (BCT) [7,11,25,30,35,43] and the edge crush test (ECT) [44,45,46,47,48]. In the ECT, four methods, contingent on the specimen shape, have been constituted. The first, called the edge clamping method [49] makes use of a specimen with the dimensions 50 mm × 50 mm, regardless of the board type. In the second, the edge-reinforced method [50,51], unsupported samples with waxed edges are tested, and their height is specifically customed for the common flute types A, B, and C. The samples are less restrained than in the previous test, and thus, in this case, the lower ECT values may occur. Another common compression test, called the neck-down method [52], utilizes the specimens with narrowed width in the middle, thus concentrating the stress there. Comparing to other methods, it might give the highest ECT value for lightweight boards. As the last procedure of ECT, the rectangular test specimen method can be mentioned [53,54]. Considering the correlation between the specimen height and ECT, it has been observed that ECT values decrease as specimen height increases, due to the sample buckling rather than sample compression [55]. The discussion of certain aspects of ECT testing, including specimen height, test duration, and fixture-clamping effects was presented in [56], and the influence of reduction in sidewall surface cutouts on ECT was presented in [57]. For gathering the measurement data from the outer surface of the specimen, the non-contact optical measurement method, e.g., video extensometry [44,58], can be used.

Despite the numerous, advanced, constantly developed methods of determining the material parameters of the cardboard, there are situations when it is a really challenging task. In such cases, solutions based on artificial intelligence (AI) turn out to be a remedy. AI can be defined as the simulation of human intelligence processes by machines, in particular by computer systems, basing on three main processes, namely, learning, reasoning and self-correction. A prime example of AI is deep learning providing the possibility of mimicking a human brain’s neural network, using an artificial neural network (ANN). ANN is a non-linear statistical model that demonstrates a complex relationship between input and output to discover a new pattern. ANN’s architecture comprises an input layer, one or more hidden layers and an output layer and works as feedforward or feedback neural network [59].

Among the numerous applications of ANN one can mention a few, e.g., medical diagnosis [60,61], image recognition [62], economics [63], biosystem engineering [64] or automobile guidance systems [65]. ANN is also utilized in the paper industry. An approach based on the machine learning intended for evaluating the effects of refining on the fibers morphology was discussed in [66], where ANN was applied and trained with experimental data to prognosticate the fibers length as a function of refining process variables. The prediction of paper characteristics, namely, apparent density, breaking length and tear resistance, based on refined chemical pulp properties using the neural network approach was demonstrated in [67,68]. A multilayer perceptron model to predict the laboratory measurements of paper quality while applying the instantaneous state of the papermaking production process has been described in [69]. The results of ANN application for the pulping process control can be found in [70,71]. To the best knowledge of the authors, no work concerning the application of ANN for estimation of mechanical parameters of corrugated board has been presented.

The objective of the presented paper is applying machine learning algorithms to create a relationship between the mechanical parameters of papers used for the production of corrugated board, its geometry and the resistance of a cardboard sample to edge crushing. Comparing the analytical, empirical or advanced numerical models described in the literature, which are used to estimate the compression resistance of cardboard across the flute, with the approach presented in the article, one can conclude that it is not only much less demanding in terms of implementation than other models, but it is as accurate and precise.

As is well known, AI can, in a similar way to the human brain, find relationships between certain parameters or features of an object or assign it to a certain group of objects. This is especially helpful when these relationships cannot be clearly described mathematically or when the set of data is incomplete, which is very common in practice. In large laboratories that deal with quality control, simple empirical models are usually used. The voices of scientists and their advanced analytical or numerical models do not reach practitioners who are looking for simple and reliable procedures. This article fills the gap. The advanced machine learning tools included here are increasingly commercially available. The presented procedure shows how to use these methods and what should be the protocol of laboratory tests in order to correctly estimate the ECT parameter of corrugated board based on an incomplete set of parameters of component papers.

## 2. Materials and Methods

### 2.1. Paperboard and Cardboard Laboratory Tests

**Figure 1:**
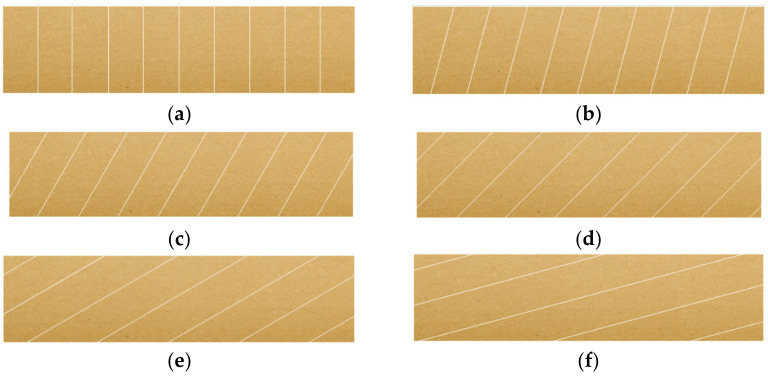
ECT samples cut at different angles: (**a**) 0 degrees (CD); (**b**) 15 degrees; (**c**) 30 degrees; (**d**) 45 degrees; (**e**) 60 degrees; (**f**) 75 degrees.

Typical component papers and six popular types of corrugated cardboard were used for the research carried out in the accredited laboratory in Aquila Września. The aim of the research was first to thoroughly examine the papers that are later used in the production of selected cardboards, and then to examine the cardboards samples in the edge crush test (ECT). Corrugated board samples are usually tested only in the CD direction, i.e., across the direction of the flute and, at the same time, across the direction of the cellulose fibers in paper. In corrugated board, the direction of the fibers in all flat and corrugated layers coincides with the direction of the wave, which results from the production process of both paper and corrugated board. However, the cardboard used for packaging is not always loaded in the CD direction only; therefore, in this study, the samples are cut and tested at different angles to the CD direction (see Figure 1).

Finally, six different directions of loading the cardboard samples were selected, i.e., classically in the CD direction, and samples were cut at 15, 30, 45, 60 and 75 degrees. In the conducted research, the testing of samples cut at an angle of 90 degrees was abandoned due to the very unreliable measurement results. This is due to the fact that in the MD (machine direction), i.e., rotated by 90 degrees in relation to CD, the sample transfers the load only through flat layers (liners), which, especially at the edges, are very flaccid and undergo global buckling very quickly.

**Figure 2:**
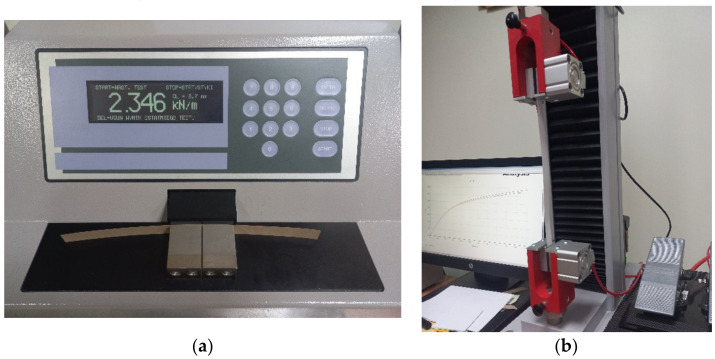
Laboratory machines and samples used in the research: (**a**) short-span compression tester for paperboard; (**b**) tensile stiffness and strength tester for paperboard.

In the first part of the research, a series of tests of all the constituent papers of each of the six selected corrugated boards was carried out. Their thickness (THK), grammage (GRM), resistance to short-span compression (SCT), and tensile stiffness (TS) were tested. All tests were carried out in accordance with the actual standards, i.e.,

- SCT according to the ISO 9895 standard (see Figure 2a);
- TS according to the EN ISO 1924-2standard (see Figure 2b).

A TMI device, model 17–36 (Messmer Büchel-Industrial Physics, LLC, Veenendaal, The Netherlands), was used to measure the SCT parameter, and the Testometric Company Ltd. (Lincoln Business Park, Rochdale, Lancashire, UK) device, was used to measure the TS parameter. Standard conditions (according to EN ISO 1924-2 standard) prevailed in the laboratory, i.e., 23 °C +/− 1 °C and relative humidity of 50% +/− 2%. Before testing, paper and paperboard samples were additionally conditioned inside an environmentally controlled laboratory with standard climatic conditions for a period of 24/48 h, as expressed by the norm ISO 187.

**Figure 3:**
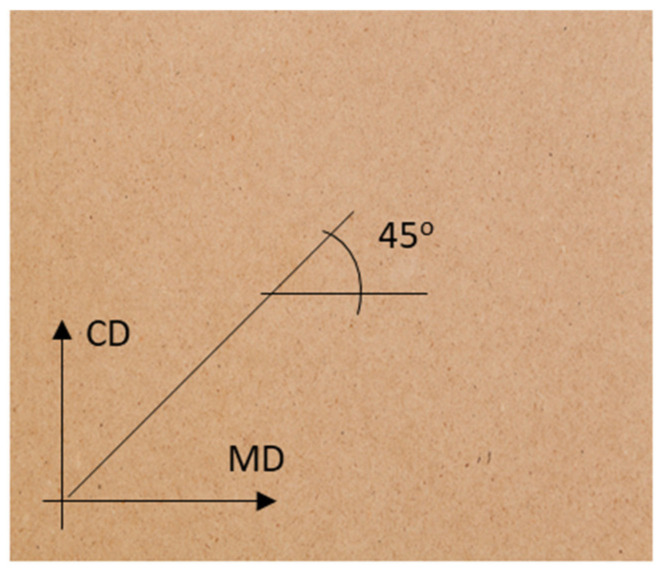
A sheet of paper with marked directions for cutting samples.

Since the paper is characterized by strong orthotropy (due to the already mentioned arrangement of fibers), the test samples were cut in two main directions of orthotropy, i.e., machine direction (MD) and transverse direction (CD). In addition, samples were also cut at an angle of 45 degrees (see Figure 3).

**Figure 4:**
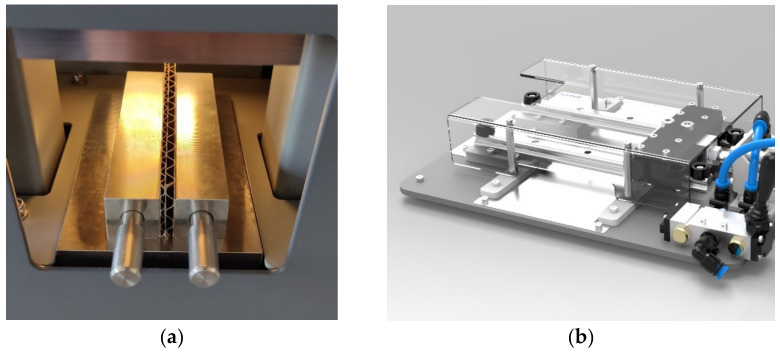
Laboratory machines and samples used in the research: (**a**) edge crush tester for corrugated board; (**b**) device to cut corrugated samples for ECT.

In the second part of the research, the resistance of corrugated cardboard to edge crushing was measured, this parameter is commonly referred to as ECT. In this test, it is extremely important to correctly cut the samples while maintaining the parallelism of both cut edges. For professional cutting, a FEMAT CUT-19AP device was used (see Figure 4b). The device for measuring the ECT parameter used in these studies was also manufactured by FEMAT (Poznań, Poland), model ECT-10-21 (see Figure 4a). Additionally, in the case of these tests, standard conditions, as well as sample preconditioning according to the ISO 187 standard, were maintained.

All test characteristics of corrugated board, i.e., declared grammage, flute type, catalog characteristics of each flute (width, height and take-up factor) as well as the main parameters of the composite papers are listed in Table 1. In order to standardize the records of test results, from now on, each cardboard is described with a wave symbol and grammage; for instance, cardboard with a BE flute and a grammage of 590 g/m^2^ is marked BE-590.

### 2.2. Artificial Neural Networks–Training Data

The goal of this work is to use artificial intelligence to find the relationship between the parameters of the component papers together with the geometry of the corrugated board and resistance to edge crushing of cardboard samples cut at different angles relative to the CD. For this purpose, traditional feedforward ANNs were used here. The training data was split into two sets because the length of the training vector is different for 3-ply and 5-ply cardboards.

Both vectors are shown in Table 2 and Table 3. Thus, the training data for the 3-ply cardboard model is a vector which has 28 elements, i.e., in order, the SCT values measured in CD, MD and 45 degrees, the TS values measured in CD, MD and 45 degrees, the wave characteristic (only for corrugated layers) as well as the thickness and grammage of all papers included in a given board. The last parameter is the angle at which the sample was rotated in the ECT. The corresponding target of the training pair is a scalar, namely, the ECT parameter of the sample cut in the corresponding direction. Therefore, the training pair is a 28-element input vector and a 1-element target value vector.

The training data for the ANN model of 5-ply cardboard is a vector consisting of 47 elements, arranged in the same way as in the ANN model of the 3-ply corrugated board (see Table 3).

All inputs were pre-scaled using a normalization procedure, where each row of training data has a mean of 0 and a standard deviation of 1. Both neural models have the same number of neurons in the hidden layer as the number of input neurons, i.e., 28 for the 3-layer cardboard model and 47 for the 5-layer corrugated board model.

The training data was divided into 3 sets: (a) training—80% of the dataset; (b) testing—10% of the dataset; (c) validating—10% of the dataset. In the process of collecting experimental data, 20 measurements of all parameters were recorded, but it was not possible to link them to specific measurements of cardboard edge crushing strength; therefore, training pairs were selected in a way that ensured a statistical distribution of all obtained parameters. This means that instead of the mean value of the individual parameter obtained from paper strength measurement, one specific result was randomly selected and assigned to the input vector, and the other input parameters were selected in a similar way. The ECT values were also drawn stochastically as the corresponding element of the training pair. This approach not only led to an increase in the number of training pairs, but it also meant that that the variability of paperboard material parameters as well as the measured ECT of the cardboard were taken into account.

To train both neural networks, a specific variant of the quasi-Newton method was used, namely, the Levenberg–Marquardt algorithm [73], which was designed to achieve second-order convergence speeds without the need to compute the Hessian matrix. When the objective function is in the form of a sum of squares (here, the mean squared normalized error was used in both model as the performance function), then the Hessian matrix can be approximated as:

(1) $$H=J^{T}J$$

and the gradient can be computed as

(2) $$g=J^{T}e$$

where J is the Jacobian matrix that contains first derivatives of the network errors with respect to the weights and biases, and e is a vector of network errors. The Jacobian matrix is usually computed using the standard backpropagation technique [74], which is much less laborious and time consuming than computing the Hessian matrix.

The Levenberg–Marquardt algorithm, similar to Newton’s method, only uses an approximation to the Hessian matrix as follows:

(3) $$x_{k+1}=x_{k}-{\left[J^{T}J+\nu I\right]}^{-1}J^{T}e,$$

where $x_{k+1}$ and $x_{k}$ are up-to-date and current vectors, respectively, where all the weights and biases of the ANN model are gathered, ν is the scaling factor and **I** is the identity matrix.

When the scalar ν is zero, the analysis step follows Newton’s method, which uses an approximate Hessian matrix. As ν increases, the analysis step is performed in the direction of the steepest gradient descent with a very small step size. As is known, near the minimum of the error function, Newton’s method is faster and more accurate; hence, the goal is to switch the algorithm towards Newton’s method as soon as possible. By decreasing ν after each successful step (when the objective function decreases) and increasing it only when a given step would increase the objective function, it efficiently switches between the two directions. In this way, the objective function at each iteration of the algorithm is always reduced.

The Levenberg–Marquardt algorithm is described in [73], while the application of this method for training neural networks is described in [74,75]. This algorithm is probably the fastest method of training medium-sized feedforward neural networks (up to several hundred weights). Therefore, it is well suited for the type and size of the network used in this study.

### 2.3. Gaussian Processes

Gaussian processes can be used as a supervised learning technique for both classification and regression tasks. The GP can be also successfully used in topology optimization [76], to solve model reduction problems [77] or inverse homogenization design [78]. The main advantage of applying the Gaussian processes to solve the regression problems is that they provide confidence measures for the predictions made. For example, in the context of prediction of the ECT value, the Gaussian processes can be used to decide which regions of the parameter space should be prioritized, based on the uncertainty of the resulting predictions. The Gaussian process can be treated as a Gaussian distribution, but not over variables rather over functions (i.e., over functions treated as infinitely long vectors containing the value of the function for each argument).

The Gaussian process is fully defined by the mean function µ such that µ(x) is the mean of f(x) and the covariance/kernel function k such that $k\left(x_{i},\;x_{j}\right)$ is the covariance between the value of the function at $x_{i}$, $f\left(x_{i}\right)$ and the value of the function at $x_{j}$, $f\left(x_{j}\right)$. Without going into details, because there are many textbooks and books on this subject, in recent scientific papers, one can also find details of their implementation [61,64]; the most important thing from the point of view of regression accuracy is the proper selection of kernel functions and proper training of hyperparameters.

The covariance function should have the following properties:

- Be symmetrical. That means that $k\left(x_{i},\;x_{j}\right)=k\left(x_{j},x_{i}\right)$;

- Be positively defined. That means that the kernel matrix $K_{xx}$ induced by k for any set of inputs should be a positive definite matrix.

As a covariance function can be treated as any function that generates a specific non-negative covariance matrix, the argument for this function is an ordered set of vectors $\left(x_{1},\ldots ,x_{N}\right)$. For instance, it can be a stationary, non-isotropic squared exponential covariance function k(x,x′), expressed by the following formula:

(4) $$k\left(x,x^{\prime }\right)=\nu \exp\left(-\frac{1}{2}\overset{M}{\underset{i}{\sum }}\omega_{i}{\left(x_{i}-x_{i}^{’}\right)}^{2}\right)+b\;,$$

where ν denotes the vertical scale of the process, while b is a bias that represents the vertical offset of the Gaussian process. The $\omega_{i}$ parameters are related to a different distance measure in each dimension. The parameter $\omega_{i}$ has a little effect on the input if it is small. Therefore, the *i*-th input is down-weighted. The hyper-parameters are directly associated with the model sensitivity in terms of the inputs. Therefore, they are of a great significance and can provide an individual measure of importance for each input of the model.

It is possible to make predictions of the new input vectors when the covariance function is already defined. However, it is necessary to determine the hyper-parameters, $r=\left[\nu ,\omega_{1},\ldots ,\omega_{M},b,\beta \right]$, before the predictions will be possible. It is possible to find the unknown parameters using any optimization procedure. In the literature, one can find methods, in which a searching for the most probable set is performed by maximizing the log likelihood function [61,64] using any gradient-based optimization algorithms, e.g., a first-order batch Levenberg–Marquardt algorithm already presented in the previous section.

## 3. Results

Since the sensitivity of the model to the input data is not known a priori, in the first step, analyses were performed using all training data. In the next step, a sensitivity analysis of the pretrained models to the input data was calculated, and an attempt was made to reduce the input vector by the parameters to which the model was the least sensitive. The complete input data is presented in Table 4, while the output data is summarized in Table 5.

As already mentioned, in the Section 2.2, two distinct model’s architectures were used for the ECT estimation of 3-layer cardboard samples and 5-layer cardboard processing. The models differ only in the number of input elements, i.e., the 3-ply cardboard model has 28 input elements, while the input vector of the 5-ply cardboard model has 47 elements.

In each of these models, a set of training data (presented in Table 4 and Table 5) and three different methods based on AI were used to estimate the ECT parameter of a corrugated board sample loaded at different angles. The first method is a classic feedforward neural network (FF), the second is a deep neural network (DL) and the last method uses the Gaussian processes (GP). Each of these methods uses 360 sets of training pairs, which includes 20 sets of material parameters of various compositions of component papers, three types of single-walled cardboard, i.e., B-410, C-590 and E-480 (or three types of double-walled cardboard, i.e., BC-790, BE-600 and BE-590), and six different angles under which the samples for the ECT were cut out.

It is worth noting that both models (in three variants each) were trained using a selected set of 288 training pairs, validated using 36 pairs and tested also using 36 pairs. All these sets were randomly selected, and the same division was used in each model, and in each variant of the AI method. The results presented in the figures and tables below have been prepared only on the test set, i.e., no training or validation data is used to check the quality of the presented models. All results shown below are summarized separately for each AI method and separately for the two models.

**Figure 5:**
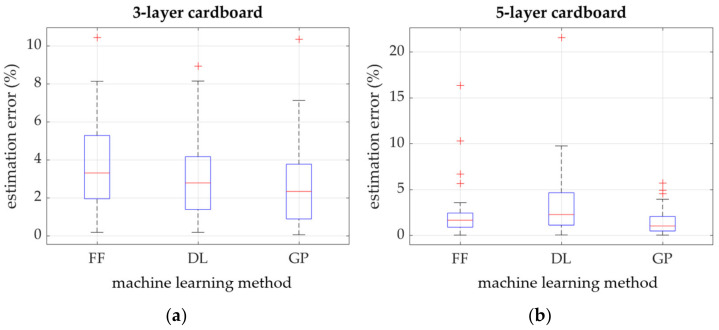
Estimation error of three different methods: (**a**) 3-layer corrugated board model; (**b**) 5-layer cardboard model.

Figure 5 shows graphs collecting the average absolute estimation error obtained using the FF, DL and GP methods in the 3-layer cardboard model (Figure 5a) and 5-layer cardboard model (Figure 5b). In the graph, bottom and top of each box are associated with the 25^th^ and 75^th^ percentiles of the obtained errors, respectively. The interquartile range is represented by the area included between the bottom and top of each box. The red line represents the median of the errors. One can notice the results skewness if the median is not centered within the box. The lines extending above and below each box are called the whiskers, and they go from the end of the interquartile range to the furthest observation within the whisker length (the adjacent value). The observations out the whisker length, the outliers, are marked with red +.

**Figure 6:**
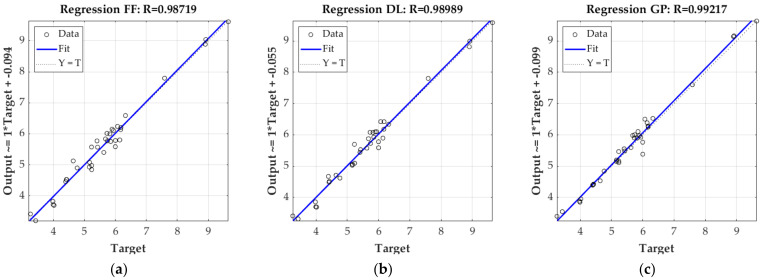
Linear regression of trained 3-ply corrugated model; (**a**) based on feedforward neural network; (**b**) based on deep neural network; (**c**) based on Gaussian process.

**Figure 7:**
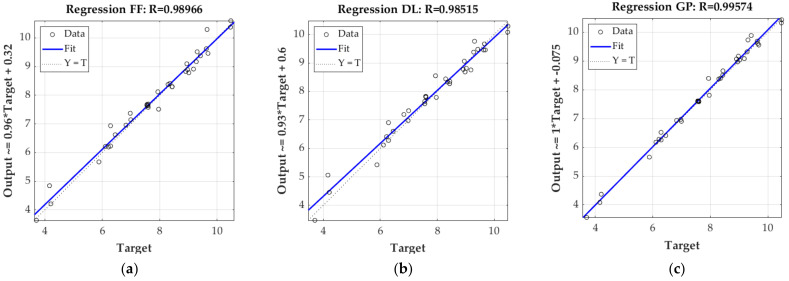
Linear regression of trained 5-ply corrugated model; (**a**) based on feedforward neural network; (**b**) based on deep neural network; (**c**) based on Gaussian process.

Figure 6 and Figure 7 show the linear regression of targets (here the ECT values) relative to outputs of each model and variant (estimated ECT values) for the testing set only. Figure 6 presents the result obtained from the 3-layer corrugated board model, while Figure 7 presents the results from the 5-layer cardboard model. In both figures, the coefficient of determination R^2^ is also shown.

**Figure 8:**
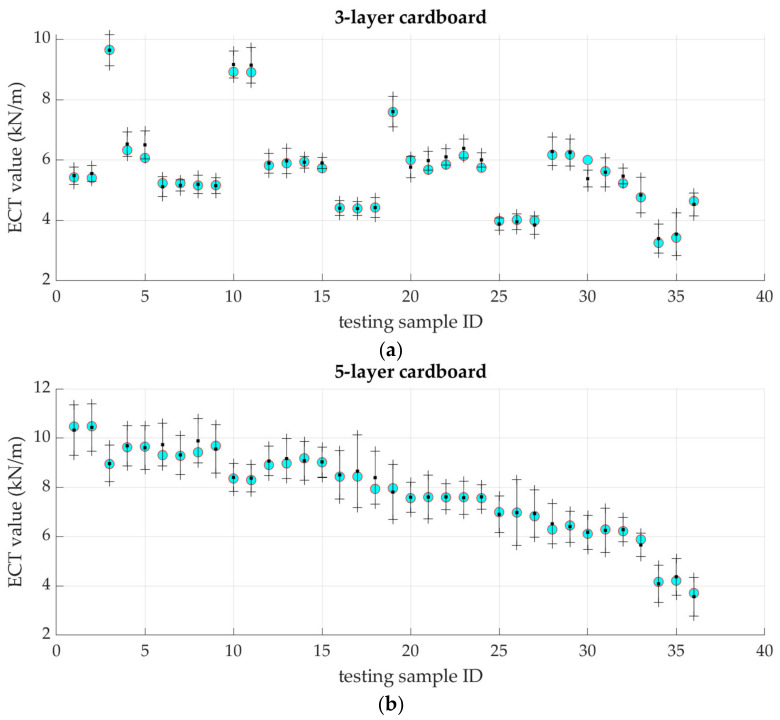
The ECT estimates using two GP-based models; (**a**) 3-ply cardboard model; (**b**) 5-ply cardboard model; the black dots represent the mean value of the prediction, while the vertical black lines represent the ±3 standard deviation for each sample. Experimental ECT values are shown as cyan dots.

Figure 8 shows the results of the estimation of the ECT parameter using the model of 3- and 5-layer corrugated model based on the Gaussian processes. In this method, the error bars are also available and are shown in Figure 8.

In the case of the GP algorithm, it is possible to obtain the sensitivity of the model to the input parameters relatively easily. It can be estimated thanks to the specific structure of the covariance matrix (see Equation (4)), where the $\omega_{i}$ parameter measures the sensitivity. The higher the value of this hyperparameter (which scales each *i*-th input parameter independently) during training process, the higher the role of this parameter in the algorithm and, therefore, the higher the sensitivity of the model to this parameter.

**Figure 9:**
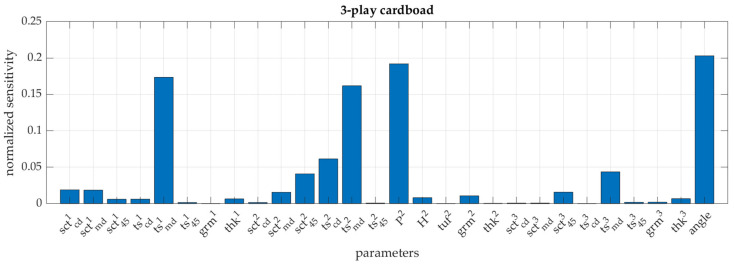
Normalized sensitivity of the GP based model (3-ply cardboard) to all 28 input parameters.

It is clearly visible in Figure 9, which presents the normalized sensitivity of the GP based model to all input parameters, that not all parameters play same important role in this model. A longer discussion on this observation is presented in the next section; therefore, only the parameters that were finally selected for further analysis, as significant, are listed here.

In the case of the 3-layer cardboard model, these are: the short-span compression strength of each paper $SCT_{CD}^{i}$ for all flat and corrugated layers of corrugated board (i.e., i=1…3), the width and height of the flute, i.e., $H_{2}$ and $P_{2}$, respectively, and the angle at which the sample is loaded in ECT. In the case of the 5-layer cardboard model, the list of parameters is slightly longer, which are again: the short-span compression strength of each paper $SCT_{CD}^{i}$ for all flat and corrugated layers of corrugated board (i.e., i=1…5), the width and height of both flutes, i.e., $H_{2}$, $P_{2}$, $H_{4}$ and $P_{4}$, and the angle at which the sample is loaded in ECT.

Table 6 summarizes the mean absolute estimation error of the ECT parameter using all the models and methods presented in this paper, including also models trained with truncated inputs vectors (marked as ‘small’). The table also shows the results obtained from other models available in the literature. Details on these additional models are presented in the discussion section. It is worth noting that for the 3-layer cardboard model, the input vector has been truncated from 28 to 6 parameters, while the 5-layer cardboard model uses a vector truncated from 47 to only 10 parameters.

## 4. Discussion

In the previous section, all the results of the laboratory testing campaign were presented, both for component papers (see Table 4) and selected corrugated board (see Table 5). In addition, the results of numerical analyses based on machine learning algorithms for estimating the resistance of corrugated board to edge crushing are also shown (see Figure 5, Figure 6, Figure 7 and Figure 8). The results presented in Figure 5 clearly show that estimations, while using the 3-ply board model based on all three machine learning methods, have an average absolute prediction error of 2.2–3.9%. On the other hand, the 5-ply cardboard model learned using all three methods based on AI have an estimation error of 1.3–3.1%. Machine learning based on the Gaussian processes performed best in both models, while neural networks, using shallow and deep learning performed, slightly worse.

The differences between various learning methods are particularly visible in Figure 5, where graphs are presented in the form of whisker plots, in which outliers are marked with red “+” symbols. However, in the 3-layer cardboard model, in all three learning methods, the maximum error reached about 9–10 percent; in the case of the 5-layer corrugated board model, the maximum error for shallow neural networks reached over 15%, and in the case of deep neural networks, it reached slightly over 20%. This does not mean, however, that the models are not optimistic; on the contrary, obtaining such good results with such a small training sample is very promising.

Figure 9 shows the normalized sensitivity of the 3-ply cardboard model to all 28 input parameters. Sensitivity is obtained for free during machine learning using GP, as described in the previous section. It is evident that not all of these parameters are equally important for the correct estimation of the ECT values. It is clear that the sensitivity of the model to the parameters, $TS_{MD}^{i}$, for each component paper (i.e., i=1…3), the wavelength of the corrugated layer ($P^{2}$) and the direction of the load (angle) are preferred by the GP learning algorithm in this case.

It is also known that the compressive and tensile strength and stiffness of the paper are closely correlated. This means that as the $SCT_{MD}$ parameter, which is the compressive strength in the MD, increases the $SCT_{CD}$, and, e.g., $TS_{45}$ parameters will also increase. Since the $SCT_{CD}$ parameter is the easiest and most frequently tested in the paperboard laboratories, it was decided to use this parameter instead of TS_MD for each component paper. In addition, the height of the corrugated layer was added to the set of learning parameters, although the model does not show high sensitivity to this model. A similar set of parameters was selected for the 5-ply cardboard model. The results presented in Table 6 clearly show that the decrease in accuracy of models trained with the truncated vectors by all three machine learning procedures is very small. The average estimation error for the 3-ply board model ranges from 2.3 to 3.9% and for the 5-ply board model from 1.9 to 2.9%.

The results obtained in this work are compared with the results available in the literature, and they are also shown in Table 6. In the last paper, Garbowski et al. [72] proposed a simplified analytical and empirical model. This paper also presents the results of the ECT estimation using a numerical model based on the finite element method as well as the results obtained while applying an empirical model. It can be seen that the accuracy of all models presented in this paper is close to the accuracy of the analytical–empirical model and numerical models presented in article [72]. It is worth noting that in the work [72], the authors did not analyze cases of loading the sample in the ECT at other angles, i.e., 15–75 degrees; only standard test in the CD was analyzed there. Therefore, the models and learning methods presented here, albeit with similar estimation precision, seem to be more universal than the analytical model, which must be extended to take into account different sample load angles in the ECT. The models proposed here do not require complicated numerical modeling (as presented, e.g., in [45]) and require only a few parameters, i.e., the basic material parameters of the component papers and the geometry of the corrugated layers are sufficient for their correct definition.

## 5. Conclusions

This paper presents the use of three different methods based on AI to solve regression problems of two different models of corrugated board: single-wall and double-wall. Although the methods are different, they are characterized by similar effectiveness and accuracy in estimating the load capacity in ECT based solely on the parameters of the board components, the geometry of the corrugated layers and loading angle. The accuracy measured on the test set only, i.e., on the set never used in the learning process in any of the models using three AI-based methods, was not lower than 96%. Similar accuracy was obtained on data truncated to just a few key parameters. This means that the presented here methods can effectively compete with advanced analytical models or with demanding numerical models and can be successfully used for ECT estimation. In order to correctly determine the edge crush resistance of corrugated board, only the compressive strength of the component papers, the geometry of the corrugated layer and the angle (if the loading direction is different than 0 degrees, i.e., CD) are necessary.

Most paperboard and cardboard laboratories have a huge database of both paper and board, and not all of them have properly working ECT virtual models. Therefore, the approach based on AI presented in this paper may be perfectly applicable in such a situation. With a large amount of training data available, these algorithms can be even more reliable, accurate and versatile.

## Figures and Tables

**Table 1:** Component papers and geometry of the corrugated boards [72].

Corrugated Board			Component Papers			Corrugated Layers		
Wave	Grammage	Height	Paper ID	Grammage	Thickness	Height	Period	Take-Up
Type	(g/m^2^)	(mm)		(g/m^2^)	(mm)	(mm)	(mm)	Factor
B	410	2.912	TL3125	124 ± 6	0.27 ± 0.1	-	-	-
			WS120	118 ± 6	0.25 ± 0.1	2.55	6.34	1.337
			TL3125	126 ± 6	0.27 ± 0.1	-	-	-
C	590	4.110	KLB170	168 ± 8	0.36 ± 0.2	-	-	-
			S.C.175	176 ± 8	0.36 ± 0.2	3.63	7.95	1.427
			KLB170	169 ± 8	0.36 ± 0.2	-	-	-
E	480	1.586	TLWC160	158 ± 8	0.17 ± 0.1	-	-	-
			WS135	133 ± 6	0.13 ± 0.1	1.16	3.50	1.236
			TLW160	159 ± 8	0.17 ± 0.1	-	-	-
BC	790	6.740	KLB170	168 ± 8	0.28 ± 0.2	-	-	-
			W135	136 ± 6	0.25 ± 0.1	2.55	6.34	1.337
			WS80	79 ± 4	0.22 ± 0.1	-	-	-
			WS135	133 ± 6	0.25 ± 0.1	3.63	7.95	1.427
			KLB170	172 ± 8	0.28 ± 0.2	-	-	-
BE	600	4.150	TLW140	141 ± 7	0.28 ± 0.2	-	-	-
			WS95	94 ± 5	0.22 ± 0.1	2.55	6.34	1.337
			WS80	81 ± 4	0.22 ± 0.1	-	-	-
			WS95	94 ± 5	0.22 ± 0.1	1.16	3.50	1.236
			TL3125	124 ± 6	0.28 ± 0.2	-	-	-
BE	590	4.120	TL3125	125 ± 6	0.32 ± 0.2	-	-	-
			WS95	94 ± 5	0.24 ± 0.1	2.55	6.34	1.337
			W80	79 ± 5	0.22 ± 0.1	-	-	-
			WS95	96 ± 5	0.24 ± 0.1	1.16	3.50	1.236
			TL3125	124 ± 6	0.29 ± 0.2	-	-	-

**Table 2:** Variable number in the input vector in the ANN model of 3-ply corrugated board.

Layer	SCT			Tensile Stiffness			Flute			GRM ^*^	THK ^*^	ANG ^*^
	CD	MD	45 d	CD	MD	45 d	Width	Height	TUF ^*^			
Liner	1	2	3	4	5	6	-	-	-	7	8	-
Flute	9	10	11	12	13	14	15	16	17	18	19	-
Liner	20	21	22	23	24	25	-	-	-	26	27	-
-	-	-	-	-	-	-	-	-	-	-	-	28

* where: TUF—wave take-up factor; GRM—grammage; THK—thickness; ANG—angle.

**Table 3:** Variable number in the input vector in the ANN model of 5-ply corrugated board.

Layer	SCT			Tensile Stiffness			Flute			GRM ^*^	THK ^*^	ANG ^*^
	CD	MD	45 d	CD	MD	45 d	Width	Height	TUF ^*^			
Liner	1	2	3	4	5	6	-	-	-	7	8	-
Flute	9	10	11	12	13	14	15	16	17	18	19	-
Liner	20	21	22	23	24	25	-	-	-	26	27	-
Flute	28	29	30	31	32	33	34	35	36	37	38	
Liner	39	40	41	42	43	44	-	-	-	45	46	
-	-	-	-	-	-	-	-	-	-	-	-	47

* where: TUF—wave take-up factor; GRM—grammage; THK—thickness; ANG—angle.

**Table 4:** The input training data for all models. Short-span compression strength as well as tensile stiffness are presented as mean values supplemented with corresponding standard deviations.

Grade ID	Paper ID	Short-Span Compression Strength						Tensile Stiffness					
		CD		MD		45 Deg		CD		MD		45 Deg	
		(kN/m)						(kN/m)					
	TL3125	2.14	±0.12	3.97	±0.05	2.92	±0.05	373.3	±2.1	1012.7	±6.3	572.7	±10.8
B-410	WS120	2.09	±0.07	4.09	±0.15	3.14	±0.08	365.1	±8.8	1024.6	±8.5	516.5	±9.7
	TL3125	2.09	±0.11	4.05	±0.11	3.11	±0.14	381.2	±11.2	1058.3	±9.8	595.1	±10.5
	KLB170	3.28	±0.18	5.96	±0.23	4.43	±0.19	527.8	±9.1	1472.1	±20.1	929.1	±20.3
C-590	S.C.175	4.18	±0.19	7.47	±0.18	5.84	±0.07	686.1	±13.9	1476.1	±10.9	924.7	±48.9
	KLB170	3.19	±0.06	5.51	±0.18	4.65	±0.13	568.1	±14.8	1445.1	±31.7	956.2	±26.6
	TLWC160	2.75	±0.20	4.20	±0.15	3.49	±0.13	412.1	±6.8	1043.6	±11.1	635.0	±11.2
E-480	WS135	2.13	±0.10	4.25	±0.15	2.95	±0.07	365.0	±9.8	1067.5	±14.5	533.5	±8.5
	TLW160	2.43	±0.11	4.09	±0.13	3.21	±0.11	443.9	±1.5	1102.1	±38.6	667.0	±5.4
	KLB170	3.39	±0.12	6.14	±0.19	4.69	±0.13	618.9	±17.6	1534.2	±6.7	990.0	±17.5
	W135	2.19	±0.09	4.27	±0.10	3.09	±0.13	369.1	±11.2	1113.5	±9.8	572.4	±22.6
BC-790	WS80	1.50	±0.08	2.30	±0.09	1.91	±0.04	317.1	±4.2	699.1	±3.3	445.0	±5.8
	WS135	2.23	±0.04	4.37	±0.15	3.18	±0.06	385.9	±12.0	1147.4	±7.6	623.5	±8.0
	KLB170	3.30	±0.18	5.98	±0.32	4.66	±0.20	592.7	±8.3	1418.9	±23.6	838.2	±12.7
	TLW140	2.61	±0.13	3.92	±0.09	3.08	±0.11	506.0	±7.1	1000.0	±12.6	622.6	±11.7
	WS95	1.69	±0.09	2.99	±0.16	2.26	±0.09	331.9	±5.5	872.7	±6.0	498.6	±10.3
BE-600	WS80	1.42	±0.03	2.56	±0.17	1.88	±0.08	273.2	±3.8	812.8	±12.3	424.8	±7.2
	WS95	1.52	±0.08	3.16	±0.13	2.46	±0.06	290.7	±6.7	885.5	±18.9	508.4	±14.7
	TL3125	2.13	±0.09	3.83	±0.13	2.93	±0.10	440.6	±3.4	1082.2	±13.3	623.3	±35.4
	TL3125	2.26	±0.13	3.64	±0.08	3.06	±0.11	412.9	±11.6	961.3	±10.0	587.0	±3.4
	WS95	1.50	±0.06	2.69	±0.13	2.01	±0.09	294.3	±8.6	756.4	±13.8	427.4	±8.8
BE-590	W80	1.47	±0.08	2.34	±0.06	1.94	±0.08	343.5	±5.8	696.5	±14.5	459.8	±5.0
	WS95	1.75	±0.10	2.96	±0.18	2.15	±0.06	332.0	±4.0	854.7	±4.2	474.4	±11.0
	TL3125	2.32	±0.07	3.75	±0.06	2.87	±0.17	413.8	±4.7	883.1	±20.9	588.3	±14.5

**Table 5:** The output training data for all models. The edge crush strength a corrugated board loaded at different angles is presented as mean values supplemented with corresponding standard deviations.

Board ID	Edge Crush Resistance											
	CD (0 Deg)		15 Deg		30 Deg		45 Deg		60 Deg		75 Deg	
	(kN/m)											
B-410	5.48	±0.09	5.17	±0.12	4.40	±0.03	3.88	±0.10	3.05	±0.10	2.29	±0.13
C-590	9.68	±0.10	9.07	±0.14	7.60	±0.02	6.24	±0.12	4.83	±0.10	3.49	±0.23
E-480	6.37	±0.17	5.92	±0.08	5.99	±0.26	5.47	±0.27	5.16	±0.17	4.56	±0.18
BC-790	10.41	±0.13	9.56	±0.37	8.38	±0.29	6.96	±0.10	6.00	±0.17	4.31	±0.16
BE-600	8.95	±0.14	8.39	±0.06	7.76	±0.14	6.46	±0.18	5.66	±0.32	4.30	±0.35
BE-590	9.68	±0.10	9.07	±0.14	7.60	±0.02	6.24	±0.12	4.83	±0.10	3.49	±0.22

**Table 6:** Mean absolute estimation error of both models based on FF, DL and GP methods trained with full-length input training vector (full) and truncated input training vector (small).

	**Model**	**Mean Absolute Error (%)**	
**Method**		**3-Layer Cardboard**	**5-Layer Cardboard**
FF (full)		3.163	1.575
FF (small)		2.273	1.921
DL (full)		2.985	3.093
DL (small)		3.948	2.916
GP (full)		2.484	1.317
GP (small)		2.260	2.150
Analytical [78]		1.760	2.987
FEM-1 [78]		1.640	2.260
FEM-2 [78]		4.010	4.227
Empirical-1 [78]		3.547	10.60
Empirical-2 [78]		1.997	9.800

## Data Availability

Not applicable.
